# Association between SARS-CoV-2 infection and newly diagnosed hypertension during pregnancy: prospective, population based cohort study

**DOI:** 10.1136/bmjmed-2022-000465

**Published:** 2023-05-24

**Authors:** Anne K. Örtqvist, Maria C. Magnus, Elisabeth Dahlqvist, Jonas Söderling, Kari Johansson, Anna Sandström, Siri E. Håberg, Olof Stephansson

**Affiliations:** 1 Clinical Epidemiology Division, Department of Medicine, Solna, Karolinska Institutet, Stockholm, Sweden; 2 Department of Obstetrics and Gynecology, Visby Hospital, Visby, Sweden; 3 Centre for Fertility and Health, Norwegian Institute of Public Health, Oslo, Norway; 4 Department of Women’s Health, Division of Obstetrics, Karolinska University Hospital, Stockholm, Sweden

**Keywords:** COVID-19, obstetrics, epidemiology, hypertension

## Abstract

**Objective:**

To study the association between SARS-CoV-2 infection and newly diagnosed hypertension during pregnancy.

**Design:**

Prospective, population based cohort study.

**Setting:**

All singleton pregnancies after 22 completed gestational weeks registered in the Swedish Pregnancy Register and the Medical Birth Registry of Norway, from 1 March 2020 to 24 May 2022.

**Participants:**

312 456 individuals available for analysis (201 770 in Sweden and 110 686 in Norway), with pregnancies that reached 42 completed gestational weeks by the end of follow-up in the pregnancy registries, excluding individuals with SARS-CoV-2 infection before pregnancy and those with a diagnosis of pre-existing hypertension or onset of hypertension before 20 gestational weeks.

**Main outcome measures:**

Newly diagnosed hypertension during pregnancy was defined as a composite outcome of a diagnosis of gestational hypertension, pre-eclampsia, HELLP (haemolysis, elevated liver enzymes, low platelets) syndrome, or eclampsia, from gestational week 20 to one week after delivery. The association between SARS-CoV-2 infection and hypertension during pregnancy was investigated with a stratified Cox proportional hazard model, adjusting for maternal age, body mass index, parity, smoking, region of birth, education, income, coexisting medical conditions, previous hypertension during pregnancy, number of healthcare visits during the past year, and vaccination against SARS-CoV-2. Pre-eclampsia was also analysed as a separate outcome.

**Results:**

Of 312 456 individuals available for analysis, 8% (n=24 566) had SARS-CoV-2 infection any time during pregnancy, 6% (n=18 051) had a diagnosis of hypertension during pregnancy, and 3% (9899) had pre-eclampsia. SARS-CoV-2 infection during pregnancy was not associated with an increased risk of hypertension during pregnancy (adjusted hazard ratio 0.99, 95% confidence interval 0.93 to 1.04) or pre-eclampsia (0.98, 0.87 to 1.10). The results were similar for SARS-CoV-2 infection in all gestational trimesters and in different time periods that corresponded to dominance of different variants of the SARS-CoV-2 virus.

**Conclusions:**

This population based study did not find any evidence of an association between SARS-CoV-2 infection during pregnancy and an increased risk of hypertension during pregnancy or pre-eclampsia.

**WHAT IS ALREADY KNOWN ON THIS TOPIC**

- Previous studies have suggested that SARS-CoV-2 infection in pregnancy is a risk factor for newly diagnosed hypertension during pregnancy, including pre-eclampsia
- Covid-19 disease is a spectrum of signs and symptoms in pregnant individuals that might resemble pre-eclampsia
- Any associations between SARS-CoV-2 infection and hypertension during pregnancy could reflect closer monitoring and a higher probability of being tested for SARS-CoV-2 infection in those with early symptoms of hypertensive disorders in pregnancy

**WHAT THIS STUDY ADDS**

- Although SARS-CoV-2 infection in pregnancy is a risk factor for several adverse pregnancy outcomes, no evidence of an association between SARS-CoV-2 infection and newly diagnosed hypertension during pregnancy was found

**How this study might affect research, practice, or policy**

- In this study, timing of infection and onset of hypertension during pregnancy was accounted for, which has been lacking in previous studies
- As SARS-CoV-2 in pregnancy is related to other adverse outcomes in pregnancy, vaccination of pregnant women is still recommended

## Introduction

Newly diagnosed hypertension during pregnancy after 20 gestational weeks includes gestational hypertension and pre-eclampsia, affects about 6-8% of the pregnant population worldwide, and is a leading cause of maternal and perinatal morbidity and mortality.^1 2^ Current knowledge suggests that pregnancy does not increase susceptibility to the SARS-CoV-2 virus, but might worsen the manifestations of covid-19 compared with non-pregnant individuals of the same age.^3^

Severe covid-19 disease has been shown to have multiorgan effects and symptomatology, similar to those seen in individuals with hypertension during pregnancy. These effects include newly diagnosed hypertension, kidney injury, liver involvement, haematological complications, pathological placentas, and endothelial dysfunction, and therefore a biological link between infection by the SARS-CoV-2 virus and hypertension during pregnancy, specifically pre-eclampsia, has been suggested.^4–6^ A recent systematic review and meta-analysis showed that SARS-CoV-2 infection during pregnancy was associated with a significant increase in the odds of developing pre-eclampsia (pooled adjusted odds ratio 1.58, 95% confidence interval 1.39 to 1.80; P<0.001).^6^ The renin-angiotensin system is an important regulator of placental function because it modulates proliferation of trophoblasts, angiogenesis, and blood flow. It has been shown that the SARS-CoV-2 virus binds to the angiotensin converting enzyme 2 receptor expressing maternal and fetal cells in the placenta, leading to changes in the renin-angiotensin system, which might have a role in the suggested association between SARS-CoV-2 infection and pre-eclampsia.^6^

Severe covid-19 disease and pre-eclampsia share the same risk factors: pre-existing hypertension, obesity, advanced maternal age, diabetes,^7 8^ chronic kidney disease,^9^ and systemic lupus erythematosus.^10^ These factors could contribute to unmeasured confounding and thus cause spurious associations. Another potential bias is that pregnancies with early symptoms of hypertension are monitored more closely and have a higher probability of being tested for SARS-CoV-2 than pregnancies without adverse symptoms. This surveillance bias could introduce non-random misclassification of the risk factor and reversed causation. Only one^11^ of the included studies in the meta-analysis^6^ considered the time between infection and pre-eclampsia (ie, temporality).

Population based healthcare and quality registers from Sweden and Norway have detailed information on dates of positive test results for SARS-CoV-2 infection and confirmed diagnoses of hypertension during pregnancy. In this study, we looked at the association between SARS-CoV-2 infection and subsequent hypertension during pregnancy and pre-eclampsia, taking into account temporality and confounding.

## Methods

### Study population and design

This prospective population based cohort study included all singleton pregnancies after 22 completed gestational weeks registered in the Swedish Pregnancy Register^12^ and the Medical Birth Registry of Norway^13^ from 1 March 2020 to 24 May 2022. Access to free perinatal care is similar in the two countries. With the unique personal identification number assigned to all citizens, individual data on births were linked to registry data to collect information on infection, outcomes, and other population characteristics. Online supplemental file describes the registers in more detail.

10.1136/bmjmed-2022-000465.supp1 Supplementary data

To avoid oversampling of short pregnancies towards the end of follow-up (preterm births would be registered as births whereas ongoing pregnancies would not yet be registered if delivery was after the end of follow-up), only pregnancies with the possibility to reach 42 completed gestational weeks by the end of follow-up in the pregnancy registries were included. Individuals with SARS-CoV-2 infection before pregnancy were excluded to ensure that we looked at SARS-CoV-2 infection during pregnancy with onset of hypertension after 20 weeks of gestation. We also excluded those with a diagnosis of pre-existing hypertension or onset of hypertension before 20 gestational weeks, defined according to the ICD-10 (International Classification of Diseases, 10th revision) or Anatomical Therapeutic Chemical codes listed in the online supplemental file.

**Figure 1:**
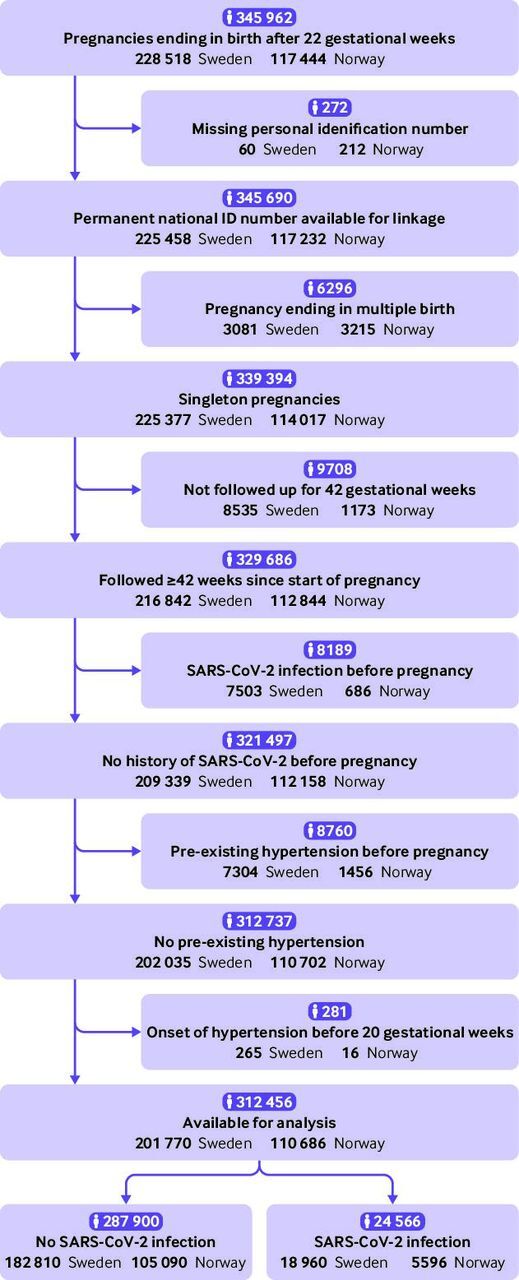
Development of cohorts for study of hypertension during pregnancy according to SARS-CoV-2 infection

**Table 1:** Participant characteristics according to SARS-CoV-2 infection during pregnancy, March 2020-May 2022, in Sweden and Norway

Characteristics*	Sweden		Norway	
	No infection (n=182 810)	SARS-CoV-2 infection (n=18 960)	No infection (n=105 090)	SARS-CoV-2 infection (n=5596)
SARS-CoV-2 vaccination:				
Vaccinated before pregnancy	5787 (3.2)	1839 (9.7)	643 (0.6)	406 (7.3)
One dose of vaccine during pregnancy	27 533 (15.1)	4284 (22.6)	15 586 (14.8)	2747 (49.1)
Not vaccinated	149 490 (81.8)	12 837 (67.7)	88 861 (84.6)	2443 (43.7)
Maternal age (years):				
<20	2046 (1.1)	149 (0.8)	902 (0.9)	36 (0.6)
20-24	18 336 (10.0)	1852 (9.8)	10 575 (10.1)	478 (8.5)
25-29	60 887 (33.3)	6396 (33.7)	35 796 (34.1)	1836 (32.8)
30-34	66 754 (36.5)	6980 (36.8)	39 055 (37.2)	2197 (39.3)
35-39	28 993 (15.9)	2972 (15.7)	15 992 (15.2)	899 (16.1)
≥40	5794 (3.2)	611 (3.2)	2770 (2.6)	150 (2.7)
Body mass index:				
<18.5	4083 (2.2)	330 (1.7)	3253 (3.1)	158 (2.8)
18.5-<25	94 922 (51.9)	9345 (49.3)	57 759 (55.0)	3066 (54.8)
25-<30	48 708 (26.6)	5375 (28.3)	23 068 (22.0)	1299 (23.2)
≥30	28 039 (15.3)	3266 (17.2)	13 765 (13.1)	737 (13.2)
Missing	7058 (3.9)	644 (3.4)	7245 (6.9)	336 (6.0)
Parity:				
0	79 398 (43.4)	7496 (39.5)	46 277 (44.0)	2092 (37.4)
1	67 885 (37.1)	7142 (37.7)	38 660 (36.8)	2061 (36.8)
≥2	35 527 (19.4)	4322 (22.8)	20 153 (19.2)	1443 (25.8)
Educational level (years):				
<9	16 812 (9.2)	1465 (7.7)	13 722 (10.1)	905 (16.2)
10-12	66 543 (36.4)	6845 (36.1)	20 138 (19.2)	1095 (9.6)
>12	89 896 (49.2)	9069 (47.8)	61 255 (58.3)	2878 (51.4)
Missing	9559 (5.2)	1581 (8.3)	9975 (9.5)	718 (12.8)
Income:				
Bottom third	46 717 (25.6)	3363 (17.7)	45 898 (43.7)	2758 (49.3)
Middle third	46 548 (25.5)	3531 (18.6)	30 324 (28.9)	1413 (25.3)
Top third	46 859 (25.6)	3220 (17.0)	23 497 (22.4)	1074 (19.2)
Missing	40 387 (22.1)	8700 (45.9)	5371 (5.1)	351 (6.3)
Birth region:				
Scandinavia	126 378 (69.1)	12 755 (67.3)	78 400 (74.6)	3613 (64.6)
Other European countries	14 574 (8.0)	1500 (7.9)	11 728 (11.2)	767 (13.7)
Middle East/Africa	28 826 (15.8)	3205 (16.9)	6987 (6.7)	764 (13.7)
Other	8518 (4.7)	661 (3.5)	7960 (7.6)	451 (8.1)
Missing	4514 (2.5)	839 (4.4)	15 (0.01)	1 (0.02)
Smoking status:				
Non-smoker	170 485 (93.3)	17 725 (93.5)	89 050 (84.7)	4678 (83.6)
Smoker	6101 (3.3)	533 (2.8)	5269 (5.0)	263 (4.7)
Missing	6224 (3.4)	702 (3.7)	10 771 (10.3)	655 (11.7)
Living with partner:				
Yes	165 110 (90.3)	17 337 (91.4)	99 197 (94.4)	5248 (93.8)
No	13 727 (7.5)	1270 (6.7)	4153 (4.0)	258 (4.6)
Missing	3973 (2.2)	353 (1.9)	1740 (1.7)	90 (1.6)
Coexisting medical conditions:				
Gestational diabetes	8184 (4.5)	846 (4.5)	6802 (6.5)	379 (6.8)
Pre-existing diabetes mellitus	1994 (1.1)	229 (1.2)	790 (0.8)	31 (0.6)
Chronic kidney disease	645 (0.4)	66 (0.3)	575 (0.6)	33 (0.6)
Systemic lupus erythematosus	259 (0.1)	30 (0.2)	88 (0.1)	2 (0.04)
History of hypertension during pregnancy:				
Yes	1413 (0.8)	112 (0.6)	3517 (3.4)	199 (3.6)
Missing	40 387 (22.1)	8700 (45.9)	0	0
No of visits to outpatient care one year before conception:				
0	87 900 (48.1)	6232 (32.9)	n/a	n/a
1-9	51 839 (28.4)	3859 (20.4)	n/a	n/a
10-29	2637 (1.4)	164 (0.9)	n/a	n/a
≥30	47 (0.0)	5 (0.0)	n/a	n/a
Missing	40 387 (22.1)	8700 (45.9)	105 090 (100.0)	5596 (100.0)
Gestational age at birth:				
Week 22+0 to 36+6	7686 (4.2)	796 (4.2)	4726 (4.5)	195 (3.5)
Week 37+0 to 39+6	83 388 (45.6)	8789 (46.4)	43 972 (41.8)	2415 (43.2)
Week ≥40+0	91 736 (50.2)	9375 (49.4)	56 392 (53.7)	2986 (53.4)

Data are numbers (percentages).

*A missing indicator was used in adjusted analyses for body mass index, education, income, birth region, smoking status, history of hypertension during pregnancy, and visits to outpatient care.

### SARS-CoV-2 infection

Data on SARS-CoV-2 infection were identified from the Public Health Agency in Sweden^14^ and the Norwegian Surveillance System for Communicable Diseases.^15^ From February 2020 onwards, reporting of all laboratory confirmed polymerase chain reaction (PCR) test results for SARS-CoV-2 was mandatory in both countries.^15 16^ In this study, individuals were defined as being infected if they had a positive PCR test result for SARS-CoV-2 between the estimated date of conception and the date of delivery. The exact date of when the PCR test was performed was used in the analysis as a proxy for onset of infection. Individuals with a positive test result for SARS-CoV-2 after the outcome of hypertension during pregnancy were treated as censored. Although limited by the absence of complete variant sequencing data, we identified four time periods when different variants of the SARS-CoV-2 virus were dominant: index (1 March 2020-31 January 2021; the first period of the pandemic with the newly identified virus before variants of the virus were identified), alpha variant (1 February 2021-30 June 2021), delta variant (1 July 2021-31 December 2021), and omicron variant (1 January 2022-24 May 2022).^17^ Testing strategies and vaccination policies for pregnant individuals in Sweden and Norway have previously been described^16 18^ and are summarised in the online supplemental file. In this study, we used information from national vaccination registries^18^ on dates and number of doses of vaccine against the SARS-CoV-2 virus.

### Hypertension during pregnancy and pre-eclampsia

Information on outcomes was collected from the Swedish Pregnancy Register, Medical Birth Registry of Norway, and national patient registers.^15 19^ Outcomes were hypertension during pregnancy, defined as having a registered ICD-10 code for gestational hypertension (O13.9), pre-eclampsia (O14.0, O14.1, and O14.9), HELLP (haemolysis, elevated liver enzymes, low platelets) syndrome (O14.2), or eclampsia (O15), from 20 gestational weeks to one week postpartum; and pre-eclampsia, which excluded gestational hypertension, but included HELLP and eclampsia. The first date of the registered ICD code was used in the analysis as the onset of outcome. Online supplemental file describes the definitions and registries used for the outcomes.

### Covariates

Population characteristics were derived from the Swedish Pregnancy Register, Medical Birth Registry of Norway, national patient registries, Statistics Sweden,^20^ and Statistics Norway.^21^ Characteristics considered potential confounders and subsequently adjusted for were maternal age at the start of pregnancy (continuous), body mass index (continuous), parity (0, 1, and ≥2), income (top, middle, and bottom thirds), region of birth (Scandinavia, other European countries, Middle East/Africa, and other, including North America, South America, Latin America, Asia, Australia, and New Zealand), highest educational level (<9, 10-12, and >12 years of schooling), smoking in early pregnancy, living with a partner, pre-existing and gestational diabetes, chronic kidney disease, systemic lupus erythematosus, history of hypertension during pregnancy in previous pregnancies, number of visits to an outpatient healthcare facility within one year of conception, and vaccination against SARS-CoV-2 (vaccinated before conception or vaccinated during pregnancy, treated as time varying). For the Norwegian analysis, information on the number of visits to an outpatient healthcare facility within one year of conception was not available.

### Statistical analysis

To investigate the association between (date of) SARS-CoV-2 infection and onset of hypertension during pregnancy and pre-eclampsia (date of diagnosis), we used a Cox proportional hazard model with gestational age as the underlying time scale and stratified by the estimated date of conception. Date of conception and gestational age at birth were estimated from the embryo transfer date, routine ultrasound assessment at about 18 gestational weeks (95%), or by using the date of the last menstrual period if other estimates were missing. The stratified Cox analysis allows for different baseline hazards for each group of pregnant individuals with an estimated date of conception on the same date, thus taking into account variations in baseline risks of SARS-CoV-2 (as well as changes in testing capacity in relation to different calendar time periods) during the course of the study. To deal with immortal time bias (ie, to avoid misclassification of time of infection caused by treating infected during follow-up as being infected during the whole follow-up) SARS-CoV-2 infection was modelled as a time varying exposure. Thus those with SARS-CoV-2 infection after the onset of hypertension during pregnancy were treated as censored. The Cox model also deals with timing of events and ensures that the test date was before the first registration of any hypertension during pregnancy.

We analysed the association between SARS-CoV-2 infection and hypertension during pregnancy, and SARS-CoV-2 infection and pre-eclampsia. In secondary analyses, we investigated SARS-CoV-2 infection by gestational trimester and time period of infection based on the dominant variant of the SARS-CoV-2 virus (index, alpha, delta, or omicron). Also, we differentiated the associations between SARS-CoV-2 infection and the risk of hypertension during pregnancy and pre-eclampsia according to whether individuals were vaccinated or not before infection with the SARS-CoV-2 virus.

Results are reported as crude and adjusted hazard ratios with 95% confidence intervals. The proportional hazards assumption was tested with Schoenfeld's residuals. We did not impute missing information on covariates because individuals with missing information, particularly for education and income, were more likely to be immigrants; limited information on immigrants is available to impute missing values. Instead, missing data were managed with a missing indicator variable in the adjusted models for the variables with missing information.

A meta-analysis, with the Knapp-Hartung random effect model, was conducted to estimate the combined adjusted hazard ratio for the primary analyses in Sweden and Norway. Heterogeneity was evaluated with Q statistics (with P values), τ^2^, and I^2^.^22^ The Swedish analyses were performed with SAS version 9.4 (SAS institute) and the Norwegian data were analysed with Stata version 15 and R version 4.1.2 (R Core Team, 2021), with the Meta package for the meta-analysis.

### Sensitivity analyses

To assess the reliability of the results, sensitivity analyses were performed: firstly, individuals with the outcome of hypertension during pregnancy, with a positive test result for SARS-CoV-2 within seven days of the outcome (before or after) were excluded from the main analysis of the association between SARS-CoV-2 infection and hypertension during pregnancy, to further reduce problems with temporality; secondly, because information on coexisting medical conditions and income were limited after 10 October 2021 in the Swedish data, the analysis was conducted with the end of follow-up as this date. The estimated parameters were then compared with estimated parameters from the main analysis where information on coexisting medical conditions and income were limited between 10 October 2021 and 24 May 2022; and thirdly, HELLP syndrome and eclampsia were excluded from the outcome of pre-eclampsia.

### Patient and public involvement

Patients and the public were not personally involved in the research process of this study, except for contributing to data in the national registries. During the study, a high priority was given to a fast study process in order to obtain results quickly for policy makers and clinicians, which precluded patient and public involvment at this point. The results have been orally presented at national conferences for clinicians, and popular scientific summaries of the findings will also be published on web pages for the pregnancy registries.

## Results

The selection of study participants is shown in figure 1. Table 1 presents the characteristics of participants according to SARS-CoV-2 infection during pregnancy, and includes a description of the proportion of missing data. Of the 312 456 individuals available for analysis (201 770 in Sweden and 110 686 in Norway), 24 566 (8%) were infected with the SARS-CoV-2 virus during pregnancy, 15% during the first trimester, 31% in the second trimester, and 53% in the third trimester (online supplemental eTable 1). Among those infected with the SARS-CoV-2 virus during pregnancy, most individuals (n=6655, 35%) in the Swedish cohort had a positive test result during the index period, followed by the omicron (n=5709, 30%), alpha (n=4855, 27%), and delta (n=1744, 9%) periods (online supplemental eTable 2). In Norway, most people (n=3211, 57%) were infected during the omicron period, followed by the delta (1221, 22%), alpha (n=590, 11%), and index (n=574, 10%) periods.

Overall, independent of SARS-CoV-2 infection, 6% of individuals available for analysis (11 620 in Sweden and 6431 in Norway) were defined as having hypertension during pregnancy, and 3% with pre-eclampsia (5891 in Sweden and 4008 in Norway) (online supplemental eTable 3). Among those infected with the SARS-CoV-2 virus, independent of infection before the outcome, 5% also had hypertension during pregnancy (1029 in Sweden and 309 in Norway) and 3% had pre-eclampsia (501 in Sweden and 186 in Norway). Mean time from SARS-CoV-2 infection any time during pregnancy to first registration of hypertension during pregnancy was 88.2 days (standard deviation 78.5, range −86 to 295) in Sweden and 57.1 days (58.0, −30 to 272) in Norway (online supplemental eTable 4). For pre-eclampsia, mean time was 86.3 days (77.8, −77 to 286) in Sweden and 60.5 days (57.6, −20 to 272) in Norway.

**Figure 2:**
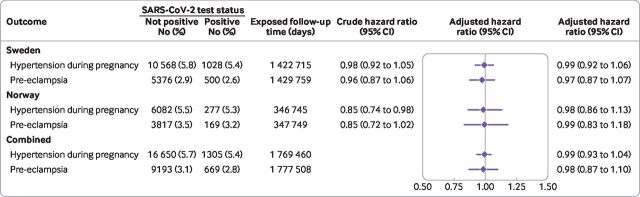
Meta-analysis of hazard ratios for hypertension during pregnancy and pre-eclampsia according to SARS-CoV-2 infection. Adjusted hazard ratios were adjusted for maternal age, body mass index, parity, income, region of birth, educational level, smoking status, living with a partner, gestational and pre-existing diabetes, chronic kidney disease, systemic lupus erythematosus, history of hypertension during pregnancy, number of visits to an outpatient healthcare facility within one year of conception (Swedish data only), and vaccination against SARS-CoV-2. Hypertension during pregnancy: heterogeneity Q=0.02 (P=0.89), τ^2^=0, I^2^=0%. Pre-eclampsia: heterogeneity Q=0.04 (P=0.85), τ^2^=0, I^2^=0%. CI=confidence interval

Figure 2 presents crude and adjusted hazard ratios and 95% confidence intervals for hypertension during pregnancy and pre-eclampsia according to SARS-CoV-2 infection in Sweden and Norway, and in Sweden and Norway combined. In the crude and adjusted analyses, we found no increased risk of hypertension during pregnancy (combined adjusted hazard ratio 0.99, 95% confidence interval 0.93 to 1.04; I^2^=0%, Q=0.02, P value for heterogeneity=0.89; τ^2^=0) or pre-eclampsia (0.98, 0.87 to 1.10; I^2^=0%, Q=0.04, P value for heterogeneity=0.85, τ^2^=0), with similar estimates for Sweden and Norway in analyses by country.

Table 2 shows crude and adjusted hazard ratios and 95% confidence intervals for hypertension during pregnancy and pre-eclampsia according to SARS-CoV-2 infection by trimester, time periods corresponding to the dominant variant of the SARS-CoV-2 virus, and vaccination status. We found no evidence for an association between hypertension during pregnancy (adjusted hazard ratio range 0.94-1.03 in Sweden, and 0.65-1.04 in Norway) and individuals infected by SARS-CoV-2 in the first, second, or third trimester. Similarly, we found no increased risk of pre-eclampsia (adjusted hazard ratio range 0.91-1.05 in Sweden and 0.77-1.04 in Norway). Also, we found no difference in the risk of hypertension during pregnancy (adjusted hazard ratio range 0.80-1.08) or pre-eclampsia (0.74-1.13) according to SARS-CoV-2 infection during time periods corresponding to the different dominant variants of the virus, or according to infection before or after vaccination for covid-19 (0.97-1.11).

**Table 2:** Hazard ratios for hypertension during pregnancy according to SARS-CoV-2 infection in different trimesters, time periods corresponding to the dominant variant of the virus, and vaccination status in Sweden and Norway

SARS-CoV-2 infection	Patients (No (%))	Follow-up time after infection (days)	Crude hazard ratio (95% CI)	Adjusted hazard ratio* (95% CI)
**Sweden: hypertension during pregnancy**				
No infection	10 568 (5.8)		Reference	Reference
Positive test result in:				
First trimester	175 (5.4)	347 634	0.98 (0.84 to 1.13)	0.94 (0.81 to 1.10)
Second trimester	364 (5.6)	725 441	0.96 (0.86 to 1.07)	0.97 (0.87 to 1.08)
Third trimester	489 (5.3)	349 640	1.01 (0.91 to 1.12)	1.03 (0.93 to 1.15)
Index variant	429 (6.5)	651 148	1.09 (0.98 to 1.20)	1.08 (0.98 to 1.20)
Alpha variant	244 (5.0)	355 105	0.93 (0.82 to 1.07)	0.91 (0.79 to 1.04)
Delta variant	73 (4.2)	130 816	0.78 (0.61 to 0.99)	0.86 (0.67 to 1.10)
Omicron variant	282 (4.9)	285 646	0.93 (0.81 to 1.07)	0.97 (0.84 to 1.11)
At least one dose of vaccine before infection	261 (5.4)	272 427	1.03 (0.89 to 1.18)	1.01 (0.87 to 1.16)
Not vaccinated	767 (5.4)	1 150 288	0.97 (0.90 to 1.05)	0.98 (0.91 to 1.06)
**Sweden: pre-eclampsia**				
No infection	5 376 (2.9)		Reference	Reference
Positive test result in:				
First trimester	90 (2.8)	348 969	0.97 (0.78 to 1.20)	0.93 (0.75 to 1.16)
Second trimester	173 (2.7)	728 416	0.91 (0.78 to 1.06)	0.91 (0.78 to 1.06)
Third trimester	237 (2.6)	352 374	1.01 (0.87 to 1.18)	1.05 (0.90 to 1.23)
Index variant	231 (3.5)	654 192	1.15 (1.00 to 1.33)	1.13 (0.98 to 1.31)
Alpha variant	120 (2.5)	356 447	0.88 (0.73 to 1.07)	0.86 (0.71 to 1.05)
Delta variant	33 (1.9)	131 436	0.73 (0.51 to 1.05)	0.79 (0.55 to 1.14)
Omicron variant	116 (2.0)	287 684	0.81 (0.65 to 1.00)	0.85 (0.68 to 1.06)
At least one dose of vaccine before infection	107 (2.2)	274 403	0.90 (0.72 to 1.12)	0.92 (0.74 to 1.14)
Not vaccinated	393 (2.8)	1 155 356	0.98 (0.88 to 1.09)	0.98 (0.88 to 1.10)
**Norway: hypertension during pregnancy**				
No infection	6082 (5.5)		Reference	Reference
Positive test result in:				
First trimester	18 (4.2)	59 065	0.66 (0.41 to 1.05)	0.65 (0.40 to 1.08)
Second trimester	69 (5.5)	143 498	0.81 (0.63 to 1.03)	0.97 (0.75 to 1.25)
Third trimester	190 (5.3)	144 182	0.90 (0.77 to 1.06)	1.04 (0.89 to 1.23)
Index variant	22 (3.9)	58 534	0.71 (0.47 to 1.09)	0.80 (0.52 to 1.24)
Alpha variant	24 (4.2)	56 803	0.80 (0.53 to 1.20)	0.95 (0.63 to 1.43)
Delta variant	60 (5.2)	97 198	0.81 (0.63 to 1.06)	0.92 (0.71 to 1.21)
Omicron variant	171 (5.8)	134 210	0.92 (0.77 to 1.09)	1.06 (0.89 to 1.27)
At least one dose of vaccine before infection	169 (5.9)	153 918	0.92 (0.77 to 1.09)	1.01 (0.85 to 1.21)
Not vaccinated	108 (4.3)	192 827	0.79 (0.65 to 0.96)	0.97 (0.79 to 1.18)
**Norway: pre-eclampsia**				
No infection	3817 (3.5)		Reference	Reference
Positive test result in:				
First trimester	13 (3.0)	59 077	0.80 (0.46 to 1.38)	0.77 (0.42 to 1.40)
Second trimester	43 (3.4)	143 792	0.79 (0.58 to 1.08)	0.94 (0.69 to 1.29)
Third trimester	113 (3.1)	144 880	0.90 (0.73 to 1.10)	1.04 (0.84 to 1.28)
Index variant	13 (2.3)	58 677	0.69 (0.40 to 1.20)	0.74 (0.42 to 1.32)
Alpha variant	13 (2.3)	56 825	0.69 (0.40 to 1.20)	0.83 (0.48 to 1.44)
Delta variant	41 (3.5)	97 415	0.89 (0.65 to 1.22)	0.99 (0.71 to 1.38)
Omicron variant	102 (3.4)	134 832	0.92 (0.73 to 1.16)	1.08 (0.85 to 1.36)
At least one dose of vaccine before infection	107 (3.7)	154 563	0.98 (0.76 to 1.22)	1.11 (0.89 to 1.38)
Not vaccinated	62 (2.5)	193 186	0.73 (0.57 to 0.95)	0.87 (0.67 to 1.14)

CI=confidence interval.

*Adjusted for maternal age, body mass index, parity, income, region of birth, educational level, smoking status, living with a partner, gestational and pre-existing diabetes, chronic kidney disease, systemic lupus erythematosus, history of hypertension during pregnancy, number of visits to an outpatient healthcare facility within one year of conception (Swedish data only), and vaccination against SARS-CoV-2.

In the sensitivity analysis excluding individuals (114 in Sweden and 40 in Norway) with SARS-CoV-2 infection within seven days of the outcome of hypertension during pregnancy, the adjusted hazard ratio was 0.91 (95% confidence interval 0.85 to 0.98) in Sweden and 0.91 (0.78 to 1.04) in Norway. Comparing estimates from Swedish data with complete data on coexisting medical conditions available up to 11 October 2021 with estimates from data with follow-up to 24 May 2022, we found no differences in the results (results not shown). When individuals with HELLP syndrome and eclampsia (176 in Sweden and 165 in Norway) were excluded from the definition of pre-eclampsia, we found only minor changes in effect estimates (results not shown) and the conclusion remained.

## Discussion

### Principal findings

Our results, based on population based cohorts from Sweden and Norway of 312 456 individuals giving birth during the first two years of the pandemic, showed no increased risk of hypertension during pregnancy or pre-eclampsia after infection with the SARS-CoV-2 virus during pregnancy. The results were similar for SARS-CoV-2 infection in any trimester, during times corresponding to the different dominant variants of the SARS-CoV-2 virus, and when adjusting for vaccination status.

### Previous studies

A recent systematic review and meta-analysis, including 28 studies,^6^ found a consistent association between SARS-CoV-2 infection and pre-eclampsia in several countries with different study designs. Discrepancies between the results from the overall meta-analysis, the individual studies included, and our study could be attributed to several factors. We controlled for a large number of confounding factors prospectively reported to national registers, thereby precluding recall bias and reducing the risks of residual confounding, whereas only about half of the studies in the meta-analysis controlled for potential confounders.

Most previous studies were limited to small study populations, with a restricted total number of participants infected with the SARS-CoV-2 virus.^6^ In the meta-analysis based on adjusted odds ratios, nine of 11 studies were cohort studies, including 8135 participants, where 36% were defined as infected with the SARS-CoV-2 virus. The other two studies,^23 24^ comprising 99% (748 526 participants of whom 1% were defined as infected with the SARS-CoV-2 virus) of the total included participants in the adjusted analysis, used a cross sectional design, making it difficult to assess whether SARS-CoV-2 infection preceded hypertension during pregnancy.

Temporality is fundamental when assessing causality.^25 26^ Only one previous study investigating the association between SARS-CoV-2 and hypertension during pregnancy took account of temporality.^11^ In that study of 249 participants, early (before 32 gestational weeks; hazard ratio 2.17, 95% confidence interval 1.11 to 4.24), but not late (1.68, 0.79 to 3.57), infection was significantly associated with hypertension during pregnancy). The authors hypothesised that participants with infection closer to term did not have a chance to develop clinical outcomes of hypertension during pregnancy. In line with this hypothesis, whether it is biologically plausible that an individual with a positive test result for SARS-CoV-2 at birth (even if the pregnant individual might have been infected some time before that) had sufficient time to develop hypertension during pregnancy is unclear. In our study, we found no difference in the results independent of whether infection occurred in the first, second, or third trimester, which makes it unlikely that our lack of association was caused by too short a time for the virus to cause hypertension during pregnancy.

Another potential reason for spurious associations is that individuals with early symptoms of hypertension during pregnancy are monitored more closely and thus have a higher probability of being tested for SARS-CoV-2 than those without hypertension during pregnancy. This practice can cause surveillance bias and non-random misclassification of infection, which could induce reversed causation. Also, pregnant individuals with severe covid-19 can develop a pre-eclampsia-like syndrome^27^ that might be distinguished from pre-eclampsia by ultrasound assessment and specific angiogenic factors,^28^ but not always from clinical symptomatology. Hence misclassification of outcome could have occurred. In a sensitivity analysis, to further study temporality and the co-occurrence of infection and hypertension during pregnancy, we excluded individuals with hypertension during pregnancy that tested positive for SARS-CoV-2 within seven days of the outcome, and found no increased risk of hypertension during pregnancy after infection.

Studies have reported that the delta variant of the SARS-CoV-2 virus could be associated with higher rates of maternal mortality^29^ and severe maternal morbidity than other variants.^30^ But another study found no significant difference in pre-eclampsia by variant type.^31^ Whole genome sequencing data of the different variants of the SARS-CoV-2 virus were missing at the population level in Sweden and Norway. When we investigated the different time periods corresponding to the dominant variants,^17^ however, we found no difference in the results.

### Strengths and limitations

A major strength of our study was the large population based cohort of prospectively collected data from two Nordic countries, allowing the analyses to account for the timing between infection and outcome, as well as controlling for potential confounders, with consistent results. We also assessed SARS-CoV-2 infection in different trimesters and included national data on vaccination against SARS-CoV-2 in our analysis. This approach is important as the vaccine will influence the probability and severity of SARS-CoV-2 infection.

This study had some limitations. Firstly, misclassification because of unidentified patients with SARS-CoV-2 infection could be a problem with this study design based on registry data. We used population based data on positive test results for SARS-CoV-2 infection verified by PCR as the risk factor, but testing capacity changed over time, and only those with severe symptoms of covid-19 were tested initially.^16^ Between June 2020 and January 2022, however, testing was available to all and recommended for even mild symptoms and in contact tracing. Also, individuals with a positive home antigen test result were strongly recommended to confirm this result with another test in the national testing system. Nevertheless, some individuals who had a positive home test result, and asymptomatic individuals who did not test, might have been included in the comparison group, which could reduce the potential associations. Also, a non-random misclassification of infection could have occurred in individuals with hypertension during pregnancy, or in individuals at high risk or with early symptoms of hypertension during pregnancy, because of more intensive testing for SARS-CoV-2 infection in these groups. Increased detection of infection or more negative test results could influence an association in both directions. With the large number of positive test results in the cohort, however, a potential true association would most likely have been seen.

Secondly, from 2020 in Sweden and Norway, proteinuria was no longer compulsory for a diagnosis of pre-eclampsia, in line with the definition of the International Society for the Study of Hypertension in Pregnancy.^32^ With the revised definition of pre-eclampsia implemented during the same time as the start of the pandemic, we chose to study hypertension during pregnancy overall and pre-eclampsia specifically, to investigate potential problems of misclassification of pre-eclampsia as gestational hypertension. A rise in incidence of pre-eclampsia after the revised definition could have caused spurious associations between SARS-CoV-2 infection and pre-eclampsia. To limit the possible effect by variation in testing capacity and change in diagnostic criteria over time, we incorporated calendar time in the models by using a stratified Cox model with estimated date of conception as strata, because this method also ensured comparisons were done in individuals at the same gestational ages at a specific calendar time. Also, when pre-eclampsia and gestational hypertension were analysed separately, the effect estimates were similar.

Thirdly, covariates with missing information were managed with a missing indicator, and in a sensitivity analysis by restricting the study period to when information on all covariates was available. Fourthly, although we adjusted for important confounders, such as body mass index, diabetes, and kidney disease, we lacked information on laboratory values and medical chart information describing the symptomatology and severity of disease of the study population. Residual and unmeasured confounding might always be present in observational and registry based studies. Lastly, although our results from two different countries gave similar results, generalisability to other populations with different test strategies and approaches to the pandemic, during a study period of new SARS-CoV-2 variants, or in pregnant individuals with primarily severe covid-19, might be limited.

### Conclusion and implications

With nationwide registry data from Sweden and Norway, we did not find any evidence of an association between SARS-CoV-2 infection during pregnancy and subsequent development of hypertension during pregnancy or pre-eclampsia. In this study, we accounted for timing of infection and onset of hypertension during pregnancy. Because infection with the SARS-CoV-2 virus in pregnancy is related to other adverse outcomes of pregnancy, vaccination of pregnant individuals in recommended.

## Data Availability

Data may be obtained from a third party and are not publicly available. Data are available by applying to the registry owners at https://helsedata.no/soknadsveiledning/ and https://www.medscinet.com/gr/forskare.aspx. No additional data available.
