# Supplementary material for: Association between SARS-CoV-2 infection and newly diagnosed hypertension during pregnancy: prospective, population based cohort study
Source: BMJ Med. 2023 May 24;2(1):e000465. doi: 10.1136/bmjmed-2022-000465 (PMC10230329; doi:10.1136/bmjmed-2022-000465)
Supplement: Supplementary data [file bmjmed-2022-000465supp001.pdf]

Online Supplement

This supplementary material has been provided by the authors to give readers additional information about their work.

Supplement to: The association between SARS-CoV-2 infection and de novo hypertension during pregnancy – a population-based cohort study

Authors: Anne K Örtqvist, Maria C Magnus, Elisabeth Dahlqvist, Jonas Söderling, Kari Johansson, Anna Sandström, Siri E Håberg, Olof Stephansson

Table of contents

Data sources and linkages in Sweden		*p. 2*
Data sources and linkages in Norway		*p. 4*
Outcome definitions		*p. 5*
Testing strategies		*p. 6*
References		*p. 7*
**eTable 1**	Participant characteristics according to SARS-CoV-2 infection in 1st, 2nd and 3rd trimester of pregnancy	*p. 8*
**eTable 2**	Participant characteristics according to SARS-CoV-2 infection during pregnancy for different time periods corresponding to the most dominating virus variant	*p. 11*
**eTable 3**	Participant characteristics according to hypertension during pregnancy and preeclampsia	*p. 14*
**eTable 4**	Time between a positive SARS-CoV-2 test and date of diagnosis of hypertension during pregnancy	*p. 17*

Örtqvist AK, *et al. BMJMED* 2024; 2:e000465. doi: 10.1136/bmjmed-2022-000465

Data sources and linkages in Sweden

The Swedish Pregnancy register (SPR)

Data in this study was provided through the Swedish Pregnancy Register (SPR). This quality register was initiated in 2013, and includes 94% of all births in Sweden (18 of 21 regions).(1) Demographical, reproductive and maternal health care data, starting at the first visit to the antenatal care clinic around the ninth gestational week, are transferred from electronic medical records within 24 hours from a reported birth. The register includes information on birth outcomes in addition to maternal background characteristics, health during pregnancy, maternal and neonatal outcomes. From this register we identified the following covariates: gestational age, parity, body mass index at start of pregnancy, smoking during pregnancy, cohabitation with partner, co-existing medical conditions and the outcome of hypertension during pregnancy.

Registers at the National Board of Health and Welfare

Covariates related to prior health conditions according to the International Classification of Diseases tenth revision (ICD-10) diagnose codes and/or Anatomical Therapeutic Chemical (ATC) codes for pre-existing hypertension and debut of hypertension up till gestational week 20, pre-existing diabetes, chronic kidney disease, and systemic lupus erythematosus, as well as number of hospital visits and stays were also collected from the National Patient Register(2) and the Prescribed Drug Register(3) (March 1, 2020-October 10, 2021) with a lookback of 3 years from March 2020.

Registers at the Public Health Agency of Sweden

From the start of the pandemic in March 2020, the SPR has been linked to the Swedish Register for Communicable Diseases (SmiNet) and the national vaccination register to retrieve regularly updated data on PCR-verified SARS-CoV-2 tests and vaccination coverage among all birthing women in the SPR.

SmiNet is the national register for communicable diseases. On February 1, 2020, SARS-CoV-2 was included in the Swedish Communicable Diseases Act, making it mandatory to report all laboratory-confirmed Polymerase Chain Reaction (PCR) cases within 24 hours to the register. From this register we used information on positive tests as well as the date of a positive PCR test. Information on key elements regarding the Swedish pandemic strategy has been discussed in detail papers by Ludvigsson JF.(4, 5)

As of January 1, 2013, healthcare providers must report all vaccinations administered within the Swedish vaccination programs to the Swedish vaccination register, held by the Public Health Agency of Sweden. The register includes type and date of all COVID-19 vaccinations. Up to end of May, 2021 in Sweden, vaccination was only recommended to pregnant women with a high risk of severe COVID-19. Thereafter, a general recommendation for pregnant women to get vaccinated was issued, although women were recommended to wait with vaccinations to after 12 weeks of gestation. As availability of vaccines was restricted initially, vaccination was still being prioritized based on age (oldest first). From August, 2021, however, the vaccine was available to all above 18 years of age. More information on COVID-19 vaccinations among pregnant and birthing women in Sweden and Norway can be found in recent publications by Örtqvist AK et al.(6) and Magnus MC et al.(7)

Registers at Statistics Sweden

Education, income (average individual income from work during 2017-2019 in tertiles) and country of birth were collected from the Total Population Register, Education Register and Income and tax register at Statistics Sweden (March 1, 2020-October 10, 2021).(8) Information on educational level and country of birth, for the period October 11, 2021-May 24, 2022, were also collected from the Swedish Pregnancy Register as this time period was not covered by the information from Statistics Sweden.

Data sources and linkages in Norway

The Emergency Preparedness Register for Covid-19
Data in this study were provided through the Emergency preparedness register for Covid-19 (Beredt C19) administered by the Norwegian Institute of Public Health, according to the Health Preparedness Act §2-4.(9) This registry was established in 2020 to provide authorities with up to date information on prevalence, causal relationships, and consequences of the Covid19 epidemic in Norway. Beredt C19 includes information already collected in the healthcare service, national health registries and administrative registers with information about the Norwegian population. The data subjects' right is safeguarded as they can contact the data controller for all different sources included in Beredt C19 in the usual way. Through Beredt C19 we used data from the following sources:

The Medical Birth Registry of Norway (MBRN)
The Norwegian Medical Birth Registry includes information on all pregnancies ending in gestational week 12 or later.(10) The registry includes information on birth outcomes in addition to maternal background characteristics, health during pregnancy, pregnancy outcomes and neonatal health.

National Patient Register
The Norwegian patient registry includes individual level information on all contacts with specialist health-care services. Information registered includes admission and discharge dates, and diagnostic codes during the hospital stay. These discharge codes are coded according to the International Classification of Diseases version 10. From this registry information on pre-existing hypertension, co-existing medical conditions and the outcomes of hypertension during pregnancy were identified.

Norwegian Surveillance System for Communicable Diseases (MSIS)
There is mandatory reporting of selected infectious diseases to this surveillance register.(11) Reporting of all Covid-19 tests is mandatory, and this register contains date of testing and test results.

The Norwegian Immunisation Register (SYSVAK)
SYSVAK is a register of vaccines in the Norwegian vaccination program, with mandatory registrations of all Covid-19 vaccinations (dates and type). Up to end of August, 2021 in Norway, vaccination was only recommended to pregnant women with a high risk of severe COVID-19. Thereafter, a general recommendation for pregnant women to get vaccinated was issued, although women were recommended to wait with vaccinations to after 12 weeks of gestation. Since January, 2022, women in Norway have been recommended vaccination regardless of trimester.(6, 7)

Statistics Norway (SSB)
Administrative data is mandatorily reported to Statistics Norway.(12) We used information from this database on household income in 2018, type of education and years of education completed by 2019.

Definition of hypertension during pregnancy (HDP) and date of diagnosis (used as proxy for disease onset) based from the national registers

HDP (excluding pre-existing hypertension or hypertension during pregnancy up until gestational week 20*) was defined by International Classification of Disease (ICD)-10 codes: O13.9, O14 or O15 identified from the national patient registries respectively (inpatient or outpatient care) in Sweden and Norway, from gestational week 20+0 up until 1 week after delivery (to identify possible postpartum preeclampsia) or the SPR and MBRN (diagnosis registered at delivery).

The following criteria was applied:
(1) one inpatient care admission, or
(2) two outpatient visits, or
(3) one outpatient visit followed by a one inpatient care admission, or
(4) one outpatient visit followed by a diagnosis in the SPR/MBRN, or
(5) a diagnosis only in the SPR/MBRN.

The date of preeclampsia diagnosis was defined as the first recording in inpatient care, outpatient care or the SPR/MBRN. Since factors (hypertension) determining diagnosis of gestational hypertension are also first signs of preeclampsia we did the following reclassification: If date of gestational hypertension occurred after diagnosis of preeclampsia this was regarded as misclassification of preeclampsia for gestational hypertension. If gestational hypertension occurred prior to diagnosis of preeclampsia we regarded this as a first indication of preeclampsia and used the date of diagnosis of gestational hypertension as date of diagnosis of preeclampsia. For women with no registration of hypertension disorders of pregnancy in the Norwegian patient registry, and the only registration was observed in the MBRN, the onset of hypertensive disorders of pregnancy was defined to be the gestational day of delivery.

***Definition of pre-existing hypertension**

Pre-existing hypertension and hypertension during pregnancy up until gestational week 20, was defined based on ICD-10 codes O10 and/or I10-I15 in the national patient registries, SPR/MBRN, and/or from Anatomical Therapeutic Chemical (ATC) codes (C02, C03, C07, C08, C09) in the Swedish Prescribed Drug Register.

Örtqvist AK, *et al. BMJMED* 2024; 2:e000465. doi: 10.1136/bmjmed-2022-000465

Testing strategies

In Sweden, SARS-CoV-2 was included in the Swedish Communicable Disease Act on 1st February, 2020, making all laboratory-confirmed polymerase chain reaction (PCR) cases of SARS-CoV-2 mandatory to report within 24 hours to SmiNet at the Public Health Agency of Sweden. Negative tests results are unfortunately not available on a national level. A non-universal population testing strategy was implemented including outpatient testing and contact tracing, starting in June 2020 and ongoing to January 2022. This type of testing mostly included symptomatic individuals, but it could also include individuals tested before and after travels, or after contact with other test-positive individuals and individuals subject to workplace testing (e.g. healthcare workers). In 23 of the 39 delivery hospitals covered by the Swedish Pregnancy Register, universal testing of all women admitted for labor or pregnancy in-patient care, independent of their current and previous medical history and COVID-19 symptoms or not, were performed.

In Norway, mandatory registration of all PCR tests for SARS-CoV-2 was implemented in the Norwegian Surveillance System for Communicable Diseases on 31st January, 2020. As Norway has not had universal testing of pregnant or delivering women, a non-universal testing strategy was operating throughout the study period. Testing was predominantly conducted on the basis of symptoms to confirm or exclude SARS-CoV-2 infection, with some additional testing conducted for particular reasons such as contact with infected persons, or mandatory testing due to travel or work. Information was available on all conducted tests, regardless of positive or negative result.

From mid-January 2022, the testing has decreased due to new recommendations. Individuals are no longer required to get tested, but to stay at home if one has symptoms. However, healthcare workers, and pregnant women with symptoms of COVID-19 in contact with health care, are still recommended to get tested, and several delivery hospitals still test pregnant women upon admission to the delivery ward independent of symptoms.

With regards to the varying testing strategies in the two countries over time, we performed a stratified cox model which compares individuals with the same estimated date of conception with each other.

References

1. Stephansson O, Petersson K, Bjork C, Conner P, Wikstrom AK. The Swedish Pregnancy Register - for quality of care improvement and research. Acta obstetricia et gynecologica Scandinavica. 2018;97(4):466-76.
2. Ludvigsson JF, Andersson E, Ekbom A, Feychting M, Kim JL, Reuterwall C, et al. External review and validation of the Swedish national inpatient register. BMC Public Health. 2011;11:450.
3. Wallerstedt SM, Wettermark B, Hoffmann M. The First Decade with the Swedish Prescribed Drug Register - A Systematic Review of the Output in the Scientific Literature. Basic & clinical pharmacology & toxicology. 2016;119(5):464-9.
4. Ludvigsson JF. The first eight months of Sweden's COVID-19 strategy and the key actions and actors that were involved. Acta paediatrica (Oslo, Norway : 1992). 2020;109(12):2459-71.
5. Ludvigsson JF. How Sweden approached the COVID-19 pandemic: summary and commentary on the national commission inquiry. Acta paediatrica (Oslo, Norway : 1992). 2022.
6. Ortqvist AK, Dahlqwist E, Magnus MC, Ljung R, Jonsson J, Aronsson B, et al. COVID-19 vaccination in pregnant women in Sweden and Norway. Vaccine. 2022;40(33):4686-92.
7. Magnus MC, Ortqvist AK, Dahlqwist E, Ljung R, Skar F, Oakley L, et al. Association of SARS-CoV-2 Vaccination During Pregnancy With Pregnancy Outcomes. Jama. 2022;327(15):1469-77.
8. Statistics Sweden. Population registers [cited 2022 Oct 4]. Available from: https://www.scb.se/vara-tjanster/bestall-data-och-statistik/bestalla-mikrodata/vilka-mikrodata-finns/.
9. Norwegian Institute of Public Health. Emergency prepardness register for COVID-19 [cited 2022 Oct 4]. Available from: https://www.fhi.no/en/id/infectious-diseases/coronavirus/emergency-preparedness-register-for-covid-19/.
10. Norwegian Institute of Public Health. Medical Birth Registry of Norway [cited 2022 18 September]. Available from: https://www.fhi.no/en/hn/health-registries/medical-birth-registry-of-norway/.
11. Norwegian Institute of Public Health. Norwegian Surveillance System for Communicable Diseases [cited 2022 Oct 4]. Available from: https://www.fhi.no/en/hn/health-registries/msis/.
12. Statistics Norway [cited 2022 Oct 4]. Available from: https://www.ssb.no/en.

Örtqvist AK, *et al. BMJMED* 2024; 2:e000465. doi: 10.1136/bmjmed-2022-000465

eTable 1. Participant characteristics according to SARS-CoV-2 infection in 1st, 2nd and 3rd trimester of pregnancy

	Sweden				Norway			
		SARS-CoV-2				SARS-CoV-2		
	**Not SARS-CoV-2**	1st trimester	2nd trimester	3rd trimester	**Not SARS-CoV-2**	1st trimester	2nd trimester	3rd trimester
**N**	182 810 (100.0)	3260 (100.0)	6480 (100.0)	9219 (100.0)	105 090 (100.0)	428 (100.0)	1257 (100.0)	3911 (100.0)
**CHARACTERISTICS**								
**SARS-CoV-2 vaccination - n (%)**								
Vaccinated prior pregnancy	5787 (3.2)	55 (1.7)	787 (12.1)	997 (10.8)	643 (0.6)	18 (4.2)	131 (10.4)	257 (6.6)
Vaccinated dose 1 during pregnancy	27 533 (15.1)	876 (26.9)	844 (13.0)	2563 (27.8)	15 586 (14.8)	86 (20.1)	396 (31.2)	2 265 (57.9)
Not vaccinated	149 490 (81.8)	2329 (71.4)	4849 (74.8)	5659 (61.4)	88 861 (84.6)	324 (75.7)	730 (58.1)	1389 (35.5)
**Maternal age (years) - n (%)**								
<20	2046 (1.1)	10 (0.3)	40 (0.6)	99 (1.1)	902 (0.9)	7 (1.6)	9 (0.7)	20 (0.5)
20-24	18 336 (10.0)	324 (9.9)	635 (9.8)	894 (9.7)	10 575 (10.1)	47 (11.0)	108 (8.6)	323 (8.3)
25-29	60 887 (33.3)	1136 (34.8)	2201 (34.0)	3061 (33.2)	35 796 (34.1)	122 (28.5)	396 (31.5)	1318 (33.7)
30-34	66 754 (36.5)	1188 (36.4)	2418 (37.3)	3372 (36.6)	39 055 (37.2)	159 (37.2)	498 (39.6)	1540 (39.4)
35-39	28 993 (15.9)	481 (14.8)	992 (15.3)	1498 (16.2)	15 992 (15.2)	82 (19.2)	201 (16.0)	616 (15.8)
>40	5794 (3.2)	121 (3.7)	194 (3.0)	295 (3.2)	2770 (2.6)	11 (2.6)	45 (3.6)	94 (2.4)
**Body Mass Index - n (%)**								
<18.5	4083 (2.2)	47 (1.4)	112 (1.7)	171 (1.9)	3253 (3.1)	10 (2.3)	31 (2.5)	117 (3.0)
18.5-<25	94 922 (51.9)	1605 (49.2)	3241 (50.0)	4501 (48.8)	57 759 (55.0)	222 (51.9)	712 (56.6)	2132 (54.5)
25-<30	48 708 (26.6)	916 (28.1)	1810 (27.9)	2648 (28.7)	23 068 (22.0)	105 (24.5)	276 (22.0)	918 (23.5)
>30	28 039 (15.3)	586 (18.0)	1092 (16.9)	1586 (17.2)	13 765 (13.1)	64 (15.0)	159 (12.7)	514 (13.1)
Missing	058 (3.9)	106 (3.3)	225 (3.5)	313 (3.4)	7245 (6.9)	27 (6.3)	79 (6.3)	230 (5.9)
**Parity - n (%)**								
Parity 0	79 398 (43.4)	1352 (41.5)	2617 (40.4)	3526 (38.2)	46 277 (44.0)	172 (40.2)	460 (36.6)	1460 (37.3)
Parity 1	67 885 (37.1)	1180 (36.2)	2458 (37.9)	3505 (38.0)	38 660 (36.8)	135 (31.2)	435 (34.6)	1491 (38.1)
Parity 2 or more	35 527 (19.4)	728 (22.3)	1405 (21.7)	2188 (23.7)	20 153 (19.2)	121 (28.3)	362 (28.8)	960 (24.6)
**Educational level (years) - n (%)**								
<9	16 812 (9.2)	224 (6.9)	477 (7.4)	763 (8.3)	13 722 (10.1)	85 (19.9)	251 (20.0)	569 (14.6)
10-12	66 543 (36.4)	1289 (39.5)	2414 (37.3)	3144 (34.1)	20 138 (19.2)	84 (9.6)	245 (19.5)	766 (19.6)
>12	89 896 (49.2)	1551 (47.6)	3177 (49.0)	4338 (47.1)	61 255 (58.3)	179 (41.8)	573(45.6)	2126 (54.4)

**eTable 1. Cont.**	**Sweden**				**Norway**			
		**SARS-CoV-2**				**SARS-CoV-2**		
	**Not SARS-CoV-2**	1st trimester	2nd trimester	3rd trimester	**Not SARS-CoV-2**	1st trimester	2nd trimester	3rd trimester
Education level, cont.								
Missing	9559 (5.2)	196 (6.0)	412 (6.4)	974 (10.6)	9975 (9.5)	80 (18.7)	188 (15.0)	450 (11.5)
**Income in tertiles- n(%)**								
Tertile 1	46 717 (25.6)	606 (18.6)	1329 (20.5)	1429 (15.5)	45 898 (43.7)	233 (54.4)	646 (51.4)	1879 (48.0)
Tertile 2	46 548 (25.5)	686 (21.0)	1587 (24.5)	1258 (13.6)	30 324 (28.9)	102 (23.8)	289 (23.0)	1022 (26.1)
Tertile 3	46 859 (25.6)	645 (19.8)	1388 (21.4)	1185 (12.9)	23 497 (22.4)	64 (15.0)	227 (18.1)	783 (20.0)
Missing	40 387 (22.1)	1302 (39.9)	2135 (32.9)	5264 (57.1)	5371 (5.1)	29 (6.8)	95 (7.6)	227 (5.8)
**Birth region - n (%)**								
Scandinavia	126 378 (69.1)	2304 (70.7)	4459 (68.8)	5992 (65.0)	78 400 (74.6)	228 (53.3)	682 (54.3)	2703 (69.1)
Other European countries	14 574 (8.0)	253 (7.8)	539 (8.3)	709 (7.7)	11 728 (11.2)	69 (16.1)	214 (17.0)	484 (12.4)
Middle East/ Africa	28 826 (15.8)	504 (15.5)	1025 (15.8)	1677 (18.2)	6987 (6.7)	91 (21.3)	244 (19.4)	429 (11.0)
Other	8518 (4.7)	115 (3.5)	219 (3.4)	325 (3.5)	7960 (7.6)	40 (9.4)	117 (9.3)	294 (7.5)
Missing	4514 (2.5)	84 (2.6)	238 (3.7)	516 (5.6)	15 (0.01)	0	0	1 (0.03)
**Smoking status - n (%)**								
Non-smoker (reference)	170 485 (93.3)	3064 (94.0)	6072 (93.7)	8588 (93.2)	89 050 (84.7)	345 (80.6)	1027 (81.7)	3306 (84.5)
Smoker	6101 (3.3)	80 (2.5)	177 (2.7)	276 (3.0)	5269 (5.0)	23 (5.4)	71 (5.7)	169 (4.3)
Missing	6224 (3.4)	116 (3.6)	231 (3.6)	355 (3.9)	10 771 (10.3)	60 (14.0)	159 (12.7)	436 (11.2)
**Living with partner - n (%)**								
Yes	165 110 (90.3)	3020 (92.6)	5964 (92.0)	8351 (90.6)	99 197 (94.4)	385 (90.0)	1163 (92.5)	3700 (94.6)
No	13 727 (7.5)	179 (5.5)	402 (6.2)	690 (7.5)	4153 (4.0)	32 (7.5)	74 (5.9)	152 (3.9)
Missing	3973 (2.2)	61 (1.9)	114 (1.8)	178 (1.9)	1740 (1.7)	11 (2.6)	20 (1.6)	59 (1.5)
**Co-existing medical conditions - n (%)**								
Gestational diabetes	8184 (4.5)	159 (4.9)	316 (4.9)	370 (4.0)	6802 (6.5)	36 (8.4)	102 (8.1)	241 (6.2)
Pre-existing diabetes mellitus	1994 (1.1)	48 (1.5)	84 (1.3)	98 (1.1)	790 (0.8)	3 (0.7)	5 (0.4)	23 (0.6)
Chronic kidney disease	645 (0.4)	12 (0.4)	26 (0.4)	28 (0.3)	575 (0.6)	2 (0.5)	8 (0.6)	23 (0.6)

**eTable 1. Cont.**	**Sweden**				**Norway**			
		**SARS-CoV-2**				**SARS-CoV-2**		
	**Not SARS-CoV-2**	1st trimester	2nd trimester	3rd trimester	**Not SARS-CoV-2**	1st trimester	2nd trimester	3rd trimester
Co-existing medical conditions, cont.								
Systemic Lupus Erythematosus	259 (0.1)	3 (0.1)	13 (0.2)	14 (0.2)	88 (0.1)	0	0	2 (0.1)
**History of hypertension during pregnancy - n (%)**								
Yes	1413 (0.8)	20 (0.6)	59 (0.9)	33 (0.4)	3517 (3.4)	10 (2.3)	47 (3.7)	142 (3.6)
Missing	40 387 (22.1)	1302 (39.9)	2135 (32.9)	5264 (57.1)	0	0	0	0
**Visits to outpatient care 1 year prior**								
0 visits	87 900 (48.1)	1181 (36.2)	2629 (40.6)	2421 (26.3)	n/a	n/a	n/a	n/a
1-9 visits	51 839 (28.4)	745 (22.9)	1644 (25.4)	1469 (15.9)	n/a	n/a	n/a	n/a
10-29 visits	2637 (1.4)	30 (0.9)	71 (1.1)	63 (0.7)	n/a	n/a	n/a	n/a
30 + visits	47 (0.0)	2 (0.1)	1 (0.0)	2 (0.0)	n/a	n/a	n/a	n/a
Missing	40 387 (22.1)	1302 (39.9)	2135 (32.9)	5264 (57.1)	105 090 (100.0)	428 (100.0)	1257 (100.0)	3911 (100.0)
**Gestational age at birth - n (%)**								
Week 22+0 -36+6	7686 (4.2)	108 (3.3)	289 (4.5)	402 (4.4)	4726 (4.5)	17 (4.0)	56 (4.5)	122 (3.1)
Week 37+0-39+6	83 388 (45.6)	1480 (45.4)	2953 (45.6)	4353 (47.2)	43 972 (41.8)	179 (41.8)	572 (45.5)	1664 (42.6)
Week >= 40+0	91 736 (50.2)	1672 (51.3)	3238 (50.0)	4464 (48.4)	56 392 (53.7)	232 (54.2)	629 (50.0)	2125 (54.3)
**Outcomes - n (%)**								
Hypertension during pregnancy	10 591 (5.8)	175 (5.4)	365 (5.6)	489 (5.3)	6122 (5.8)	18 (4.2)	69 (5.5)	222 (5.7)
Preeclampsia	5390 (2.9)	90 (2.8)	174 (2.7)	237 (2.6)	3822 (3.6)	13 (3.0)	43 (3.4)	130 (3.3)

eTable 2. Participant characteristics according to SARS-CoV-2 during pregnancy for different time periods corresponding to the most dominating virus variant

	Sweden				Norway			
	**Index**	**Alpha**	**Delta**	**Omicron**	**Index**	**Alpha**	**Delta**	**Omicron**
**N**	6655 (100.0)	4855 (100.0)	1744 (100.0)	5706 (100.0)	574 (100.0)	590 (100.0)	1221 (100.0)	3211 (100.0)
**CHARACTERISTICS**								
**SARS-CoV-2 vaccination - n (%)**								
Vaccinated prior pregnancy	0	23 (0.5)	248 (14.2)	1568 (27.5)	0	4 (0.7)	116 (9.5)	286 (8.9)
Vaccinated dose 1 during pregnancy	326 (4.9)	908 (18.7)	569 (32.6)	2481 (43.5)	7 (1.2)	62 (10.5)	420 (34.4)	2258 (70.3)
Not vaccinated	6329 (95.1)	3924 (80.8)	927 (53.2)	1657 (29.0)	567 (98.8)	524 (88.8)	685 (56.1)	667 (20.8)
**Maternal age (years) - n (%)**								
<20	42 (0.6)	28 (0.6)	25 (1.4)	54 (0.9)	2 (0.4)	7 (1.2)	15 (1.2)	12 (0.4)
20-24	698 (10.5)	480 (9.9)	171 (9.8)	503 (8.8)	59 (10.3)	66 (11.2)	104 (8.5)	249 (7.8)
25-29	2312 (34.7)	1690 (34.8)	554 (31.8)	1840 (32.2)	194 (33.8)	175 (29.7)	378 (31.0)	1089 (33.9)
30-34	2400 (36.1)	1790 (36.9)	615 (35.3)	2175 (38.1)	223 (38.9)	233 (39.5)	461 (37.8)	1280 (39.9)
35-39	1002 (15.1)	704 (14.5)	317 (18.2)	949 (16.6)	79 (13.8)	88 (14.9)	225 (18.4)	507 (15.8)
>40	201 (3.0)	163 (3.4)	62 (3.6)	185 (3.2)	17 (3.0)	21 (3.6)	38 (3.1)	74 (2.3)
**Body Mass Index - n (%)**								
<18.5	101 (1.5)	82 (1.7)	39 (2.2)	108 (1.9)	14 (2.4)	14 (2.4)	39 (3.2)	91 (2.8)
18.5-<25	3282 (49.3)	2423 (49.9)	814 (46.7)	2826 (49.5)	294 (51.2)	295 (50.0)	679 (55.6)	1798 (56.0)
25-<30	1864 (28.0)	1382 (28.5)	528 (30.3)	1601 (28.1)	148 (25.8)	135 (22.9)	273 (22.4)	743 (23.1)
>30	1182 (17.8)	810 (16.7)	299 (17.1)	975 (17.1)	75 (13.1)	96 (16.3)	159 (13.0)	407 (12.7)
Missing	226 (3.4)	158 (3.3)	64 (3.7)	196 (3.4)	43 (7.5)	50 (8.5)	71 (5.8)	172 (5.4)
**Parity - n (%)**								
Parity 0	2828 (42.5)	1896 (39.1)	686 (39.3)	2086 (36.6)	213 (37.1)	207 (35.1)	484 (39.6)	1188 (37.0)
Parity 1	2375 (35.7)	1885 (38.8)	591 (33.9)	2291 (40.2)	196 (34.2)	221 (37.5)	399 (32.7)	1245 (38.8)
Parity 2 or more	1452 (21.8)	1074 (22.1)	467 (26.8)	1329 (23.3)	165 (28.8)	162 (27.5)	338 (27.7)	778 (24.2)
**Educational level (years) - n (%)**								
<9	645 (9.7)	397 (8.2)	139 (8.0)	284 (5.0)	115 (20.0)	122 (20.7)	262 (21.5)	406 (12.6)

**eTable 2. Cont.**	**Sweden**				**Norway**			
Education level, cont.	**Index**	**Alpha**	**Delta**	**Omicron**	**Index**	**Alpha**	**Delta**	**Omicron**
10-12	2726 (41.0)	1957 (40.3)	535 (30.7)	1627 (28.5)	107 (18.6)	122 (20.7)	216 (17.7)	650 (20.2)
>12	3209 (48.2)	2286 (47.1)	774 (44.4)	2800 (49.1)	252 (43.9)	237 (40.2)	554 (45.4)	1835 (57.2)
Missing	75 (1.1)	215 (4.4)	296 (17.0)	995 (17.4)	100 (17.4)	109 (18.5)	189 (15.5)	320 (10.0)
**Income in tertiles – n (%)**								
Tertile 1	2161 (32.5)	1154 (23.8)	48 (2.8)	0	225 (39.2)	9 (40.5)	496 (40.6)	1077 (33.5)
Tertile 2	2270 (34.1)	1234 (25.4)	27 (1.5)	0	175 (30.5)	166 (28.1)	351 (38.8)	1010 (31.5)
Tertile 3	2103 (31.6)	1095 (22.6)	22 (1.3)	0	138 (24.0)	143 (24.2)	283 (23.2)	942 (29.3)
Missing	54 (0.8)	1302 (26.8)	1638 (93.9)	5706 (100)	36 (6.3)	42 (7.1)	91 (7.5)	182 (5.7)
**Birth region - n (%)**								
Scandinavia	4460 (67.0)	3462 (71.3)	993 (56.9)	3840 (67.3)	294 (51.2)	288 (48.8)	666 (54.6)	2365 (73.7)
Other European countries	585 (8.8)	372 (7.7)	169 (9.7)	374 (6.6)	80 (13.9)	107 (18.1)	255 (18.4)	355 (11.1)
Middle East/ Africa	1356 (20.4)	755 (15.6)	343 (19.7)	751 (13.2)	143 (24.9)	139 (23.6)	213 (17.4)	269 (8.4)
Other	248 (3.7)	181 (3.7)	61 (3.5)	171 (3.0)	56 (9.8)	56 (9.5)	117 (9.6)	222 (6.9)
Missing	6 (0.1)	85 (1.8)	178 (10.2)	570 (10.0)	1 (0.2)	0	0	0
**Smoking status - n (%)**								
Non-smoker (reference)	6252 (93.9)	4565 (94.0)	1601 (91.8)	5307 (93.0)	473 (82.4)	480 (81.4)	999 (81.8)	2726 (84.9)
Smoker	172 (2.6)	129 (2.7)	66 (3.8)	166 (2.9)	36 (6.3)	32 (5.4)	60 (4.9)	135 (10.9)
Missing	231 (3.5)	161 (3.3)	77 (4.4)	233 (4.1)	65 (11.3)	78 (13.2)	162 (13.3)	350 (10.9)
**Living with partner - n (%)**								
Yes	6122 (92.0)	4456 (91.8)	1576 (90.4)	5183 (90.8)	534 (93.0)	528 (89.5)	1131 (92.6)	3055 (95.1)
No	427 (6.4)	298 (6.1)	137 (7.9)	408 (7.2)	29 (5.1)	44 (7.5)	72 (5.9)	113 (3.5)
Missing	106 (1.6)	101 (2.1)	31 (1.8)	115 (2.0)	11 (1.9)	18 (3.1)	18 (1.5)	43 (1.3)
**Co-existing medical conditions - n (%)**								
Gestational diabetes	441 (6.6)	235 (4.8)	55 (3.2)	115 (2.0)	46 (8.0)	52 (8.9)	102 (8.4)	179 (5.6)
Pre-existing diabetes mellitus	84 (1.3)	64 (1.3)	20 (1.1)	61 (1.1)	6 (1.1)	2 (0.3)	6 (0.5)	17 (0.5)
Chronic kidney disease	28 (0.4)	20 (0.4)	3 (0.2)	15 (0.3)	1 (0.2)	1 (0.2)	11 (0.9)	20 (0.6)

Örtqvist AK, *et al. BMJMED* 2024; 2:e000465. doi: 10.1136/bmjmed-2022-000465

**eTable 2. Cont.**	**Sweden**				**Norway**			
Co-existing medical conditions, cont.	**Index**	**Alpha**	**Delta**	**Omicron**	**Index**	**Alpha**	**Delta**	**Omicron**
Systemic Lupus Erythematosus	9 (0.1)	10 (0.2)	0	11 (0.2)	1 (0.2)	0	0	1 (0.03)
**History of hypertension during pregnancy- n (%)**								
Yes	69 (1.0)	42 (0.9)	1 (0.1)	0	15 (2.6)	23 (3.9)	32 (2.6)	129 (4.0)
Missing	54 (0.8)	1302 (26.8)	1638 (93.9)	5706 (100)	0	0	0	0
**Visits to outpatient care 1 year prior - n (%)**								
0 visits	3972 (59.7)	2192 (45.1)	68 (3.9)	n/a	n/a	n/a	n/a	n/a
1-9 visits	2515 (37.8)	1307 (26.9)	37 (2.1)	n/a	n/a	n/a	n/a	n/a
10-29 visits	112 (1.7)	51 (1.1)	1 (0.1)	n/a	n/a	n/a	n/a	n/a
30 + visits	2 (0.0)	3 (0.1)	(0.0)	n/a	n/a	n/a	n/a	n/a
Missing	54 (0.8)	1302 (26.8)	1638 (93.9)	5706 (100)	574 (100.0)	590 (100.0)	1221 (100.0)	3211 (100.0)
**Gestational age at birth - n (%)**								
Week 22+0 -36+6	326 (4.9)	207 (4.3)	75 (4.3)	188 (3.3)	23 (4.0)	18 (3.1)	46 (3.8)	108 (3.4)
Week 37+0-39+6	3068 (46.1)	2178 (44.9)	811 (46.5)	2 732 (47.9)	234 (40.8)	274 (46.4)	548 (44.9)	1359 (42.3)
Week >= 40+0	3261 (49.0)	2470 (50.9)	858 (49.2)	2 786 (48.8)	317 (55.2)	298 (50.5)	627 (51.4)	1744 (54.3)
**Outcomes - n (%)**								
Hypertension during pregnancy	429 (6.4)	245 (5.0)	73 (4.2)	282 (4.9)	22 (3.8)	25 (4.2)	66 (5.4)	196 (6.1)
Preeclampsia	231 (3.5)	121 (2.5)	33 (1.9)	116 (2.0)	13 (2.3)	13 (2.2)	42 (3.4)	118 (3.7)

[a]The background characteristics for those without SARS-CoV-2 (Sweden: n=182 810; Norway: n=105 090) can be found in eTable 1. Overall, those with no positive test and those with a positive test in each time period, corresponds to the total population of 201 770 in Sweden and 110 686 in Norway.

eTable 3. Participant characteristics according to hypertension during pregnancy and preeclampsia

	**Sweden**			**Norway**		
	**No hypertension during pregnancy**	**Hypertension during pregnancy**	**Preeclampsia**	**No hypertension during pregnancy**	**Hypertension during pregnancy**	**Preeclampsia**
N	190 150 (100.0)	11 620 (100.0)	5891 (100.0)	104 255 (100.0)	6431 (100.0)	4008 (100.0)
**CHARACTERISTICS**						
**SARS-CoV-2 during pregnancy - n (%)**						
Yes	17 931 (9.4)	1029 (8.9)	501 (8.5)	5287 (5.1)	309 (4.8)	186 (4.6)
No	172 219 (90.6)	10 591 (91.1)	5390 (91.5)	98 968 (94.9)	6122 (95.2)	3822 (95.4)
**COVID-19 vaccination - n (%)**						
Vaccinated prior pregnancy	7124 (3.7)	502 (4.3)	231 (3.9)	975 (0.9)	74 (1.2)	50 (1.3)
Vaccinated dose 1 during pregnancy	29 922 (15.7)	1895 (16.3)	890 (15.1)	17 064 (16.4)	1269 (19.7)	780 (19.7)
Not vaccinated	153 104 (80.5)	9223 (79.4)	4770 (81.0)	82 216 (82.7)	5088 (79.1)	3169 (79.1)
**Maternal age (years) - n (%)**						
<20	2071 (1.1)	124 (1.1)	87 (1.5)	851 (0.8)	87 (1.4)	75 (1.9)
20-24	19 016 (10.0)	1172 (10.1)	671 (11.4)	10 242 (9.8)	811 (12.6)	567 (14.2)
25-29	63 288 (33.3)	3995 (34.4)	2069 (35.1)	35 433 (34.0)	2199 (34.2)	1382 (34.5)
30-34	69 750 (36.7)	3984 (34.3)	1968 (33.4)	39 105 (37.5)	2147 (33.4)	1287 (32.1)
35-39	30 122 (15.8)	1843 (15.9)	876 (14.9)	15 919 (15.3)	972 (15.1)	573 (14.3)
>40	5903 (3.1)	502 (4.3)	220 (3.7)	2705 (2.6)	215 (3.3)	124 (3.1)
**Body Mass Index - n (%)**						
<18.5	4278 (2.2)	135 (1.2)	87 (1.5)	3296 (3.2)	115 (1.8)	91 (2.3)
18.5-<25	99 903 (52.5)	4364 (37.6)	2298 (39.0)	58 146 (55.8)	2679 (41.7)	1710 (42.7)
25-<30	50 503 (26.6)	3580 (30.8)	1776 (30.1)	22650 (21.7)	1717 (26.7)	1046 (26.1)
>30	28 142 (14.8)	3163 (27.2)	1515 (25.7)	12938 (12.4)	1564 (24.3)	941 (23.5)
Missing	7324 (3.9)	378 (3.3)	215 (3.6)	7225 (6.9)	356 (5.5)	220 (5.5)
**Parity - n (%)**						
Parity 0	79 230 (41.7)	7664 (66.0)	4136 (70.2)	44 315 (42.5)	4054 (63.0)	2643 (65.9)

**eTable 3. Cont.**	**Sweden**			**Norway**		
Parity, cont.	**No hypertension during pregnancy**	**Hypertension during pregnancy**	**Preeclampsia**	**No hypertension during pregnancy**	**Hypertension during pregnancy**	**Preeclampsia**
Parity 1	72 397 (38.1)	2630 (22.6)	1158 (19.7)	39 124 (37.5)	1597 (24.8)	891 (22.2)
Parity 2 or more	38 523 (20.3)	1326 (11.4)	597 (10.1)	20 816 (20.0)	780 (12.1)	474 (11.8)
**Educational level (years) - n (%)**						
<9	17 483 (9.2)	794 (6.8)	484 (8.2)	13 681 (13.1)	946 (14.7)	654 (6.3)
10-12	68 913 (36.2)	4475 (38.5)	2270 (38.5)	19 817 (19.0)	1416 (22.0)	926 (23.1)
>12	93 123 (49.0)	5842 (50.3)	2866 (48.7)	60 496 (58.0)	3637 (56.6)	2150 (53.6)
Missing	10 631 (5.6)	509 (4.4)	271 (4.6)	10 261 (9.8)	432 (6.7)	278 (6.9)
**Income in tertiles - n (%)**						
Tertile 1	47 613 (25.0)	2467 (21.2)	1400 (23.8)	32 750 (31.4)	2238 (34.8)	1460 (36.4)
Tertile 2	47 139 (24.8)	2940 (25.3)	1462 (24.8)	32 982 (31.6)	2006 (31.2)	1234 (30.8)
Tertile 3	46 583 (24.5)	3496 (30.1)	1689 (28.7)	33 047 (31.7)	1941 (30.2)	1150 (28.7)
Missing	46 451 (24.4)	2636 (22.7)	1287 (21.8)	5476 (5.3)	246 (3.8)	164 (4.1)
**Birth region - n (%)**						
Scandinavia	129 898 (68.3)	9235 (79.5)	4493 (76.3)	76 763 (73.6)	5250 (81.6)	3232 (80.6)
Other European countries	15 330 (8.1)	744 (6.4)	369 (6.3)	11 950 (11.5)	545 (8.5)	327 (8.2)
Middle East/ Africa	31 059 (16.3)	972 (8.4)	641 (10.9)	7476 (7.2)	275 (4.3)	199 (5.0)
Other	8765 (4.6)	414 (3.6)	245 (4.2)	8050 (7.7)	361 (5.6)	250 (6.2)
Missing	5098 (2.7)	255 (2.2)	143 (2.4)	16 (0.02)	0	0
**Smoking status - n (%)**						
Non-smoker	177 358 (93.3)	10 852 (93.4)	5500 (93.4)	88 263 (84.7)	5465 (85.0)	3403 (84.9)
Smoker	6288 (3.3)	346 (3.0)	167 (2.8)	5205 (5.0)	327 (5.1)	204 (5.1)
Missing	6504 (3.4)	422 (3.6)	224 (3.8)	10 787 (10.4)	639 (10.0)	401 (10.0)
**Living with partner - n (%)**						
Yes	171 984 (90.4)	10 463 (90.0)	5238 (88.9)	98 403 (94.4)	6042 (94.0)	3749 (93.5)
No	14 047 (7.4)	950 (8.2)	521 (8.8)	4133 (4.0)	278 (4.3)	177 (4.4)
Missing	4119 (2.2)	207 (1.8)	132 (2.2)	1719 (1.7)	111 (1.7)	82 (2.1)

Örtqvist AK, *et al. BMJMED* 2024; 2:e000465. doi: 10.1136/bmjmed-2022-000465

eTable 3. Cont.	Sweden			Norway		
	No hypertension during pregnancy	Hypertension during pregnancy	Preeclampsia	No hypertension during pregnancy	Hypertension during pregnancy	Preeclampsia
**Co-existing medical conditions - n (%)**						
Gestational diabetes	8345 (4.4)	685 (5.9)	319 (5.4)	6569 (6.3)	612 (9.5)	376 (9.4)
Pre-existing diabetes mellitus	1891 (1.0)	332 (2.9)	206 (3.5)	701 (0.7)	120 (1.9)	90 (2.3)
Chronic kidney disease	655 (0.3)	56 (0.5)	35 (0.6)	555 (0.5)	53 (0.8)	34 (0.9)
Systemic Lupus Erythematosus	267 (0.1)	22 (0.2)	15 (0.3)	81 (0.1)	9 (0.1)	6 (0.2)
**History of hypertension during pregnancy - n (%)**						
Yes	1248 (0.7)	277 (2.4)	135 (2.3)	2889 (2.8)	827 (12.9)	489 (12.2)
Missing	46 451 (24.4)	2636 (22.7)	1287 (21.8)	0	0	0
**Visits to outpatient care 1 year prior**						
0 visits	88 962 (46.8)	5170 (44.5)	2610 (44.3)	n/a	n/a	n/a
1-9 visits	52 123 (27.4)	3575 (30.8)	1857 (31.5)	n/a	n/a	n/a
10-29 visits	2566 (1.3)	235 (2.0)	134 (2.3)	n/a	n/a	n/a
30 + visits	48 (0.0)	4 (0.0)	3 (0.1)	n/a	n/a	n/a
Missing	46 451 (24.4)	2636 (22.7)	1287 (21.8)	104 255 (100.0)	6431 (100.0)	4008 (100.0)
**Gestational age at birth - n (%)**						
Week 22+0 -36+6	7240 (3.8)	1242 (10.7)	1029 (17.5)	4110 (3.9)	811 (12.6)	705 (17.6)
Week 37+0-39+6	86 174 (45.3)	6003 (51.7)	3331 (56.5)	43 165 (41.4)	3222 (50.1)	2103 (52.5)
Week >= 40+0	96 736 (50.9)	4375 (37.7)	1531 (26.0)	56 980 (54.7)	2398 (37.3)	1200 (29.9)

eTable 4. Time between a positive SARS-CoV-2 test and date of diagnosis of hypertension during pregnancy and preeclampsia

**Outcome and time (days)**	**Sweden**	**Norway**
**Hypertension during pregnancy**		
Mean ± SD	88.2±78.5	57.1±58.0
Median (IQR)	74.0 (24.0-136.0)	45.5 (16.0-82.0)
Range, min-max	-86.0-295.0	-30.0-272.0
**Preeclampsia**		
Mean ± SD	86.3±77.8	60.5±57.6
Median (IQR)	70.5 (19.0-140.0)	48 (20.0-86.5)
Range, min-max	-77.0-286.0	-20.0-272.0

Örtqvist AK, *et al. BMJMED* 2024; 2:e000465. doi: 10.1136/bmjmed-2022-000465
